# SOX9 indirectly regulates CEACAM1 expression and immune resistance in melanoma cells

**DOI:** 10.18632/oncotarget.7379

**Published:** 2016-02-14

**Authors:** Shira Ashkenazi, Rona Ortenberg, Michal Besser, Jacob Schachter, Gal Markel

**Affiliations:** ^1^ Ella Lemelbaum Institute of Melanoma, Sheba Medical Center, Ramat Gan, Israel; ^2^ Department of Clinical Microbiology and Immunology, Sackler Faculty of Medicine, Tel Aviv University, Israel; ^3^ Talpiot Medical Leadership program, Sheba Medical Center, Ramat Gan, Israel

**Keywords:** CEACAM1, melanoma, SOX9, T cells, Immune checkpoint, Immunology and Microbiology Section, Immune response, Immunity

## Abstract

As melanoma cells are immunogenic, they instigate an adaptive immune response and production of anti-tumor T-cells. A central factor in this interaction is CEACAM1 (carcinoembryonic antigen cell adhesion molecule 1), a transmembrane glycoprotein previously shown in our lab to protect melanoma cells from T cell-mediated killing. In this study, we examine the role of transcription factor SOX9 in the regulation of CEACAM1 expression and immune resistance in melanoma cells. Knockdown of endogenous SOX9 results in CEACAM1 up-regulation, while its overexpression leads to the opposite effect. We show that SOX9 controls CEACAM1 expression at a transcriptional level, but in an indirect manner, as regulation of the CEACAM1 promoter remains intact even when all eight potential SOX9-binding sites are abolished. A series of promoter truncations localizes the SOX9-controlled area to the proximal 200bp of the promoter. Point mutations in putative Sp1 and ETS1 binding sites identify these transcription factors as the primary SOX9-controlled mediators. Co-immunoprecipitation studies show that SOX9 and Sp1 physically interact in melanoma cells, while silencing of SOX9 down-regulates ETS1, but not Sp1, in the same cells. Finally, knockdown of SOX9 indeed renders melanoma cells resistant to T cell-mediated killing, in line with the increased CEACAM1 expression. In conclusion, we show that SOX9 regulates CEACAM1 expression in melanoma cells, and thereby their immune resistance. As CEACAM1 is a pivotal protein in melanoma biology and immune crosstalk, further understanding of its regulation can provide new insights and contribute to the development of novel approaches to therapy.

## INTRODUCTION

Melanoma is the most common form of fatal skin cancer, being responsible for 75% of skin cancer related deaths [1]. It is an immunogenic tumor, as melanoma cells express tumor associated antigens and contain the highest DNA mutation rate [2], [3], thereby instigating an adaptive immune response and production of specific anti-tumor T cells [4]. Advancements in the understanding of tumor-immune system interactions led to the development of new therapeutic agents in the past years. Ipilimumab is a monoclonal antibody that blocks cytotoxic T lymphocyte-associated protein 4 (CTLA4), approved in 2011 by the FDA. Pembrolizumab and Nivolumab, antibodies targeting PD-1, were approved in 2014 [5]. Thus, understanding of the mechanisms involved in melanoma immune-resistance is of great importance.

The carcinoembryonic antigen cell adhesion molecule 1 (CEACAM1) is a transmembrane glycoprotein that belongs to the CEA family, encoded on chromosome 19. CEACAM1 is expressed on epithelial, endothelial, myeloid and lymphoid cells [6], and its expression mediates intercellular protein interactions and intracellular signaling. it interacts homophilically with CEACAM1 and heterophilically with CEACAM5 through its extracellular Ig-like domains [7]. CEACAM1 is subjected to alternative splicing, giving rise to two forms of cytoplasmic tail; a long form containing two immunodominant tyrosine based inhibitory motifs (ITIMs) and a short form devoid of ITIMs.

CEACAM1 has been shown to be dysregulated in several tumors, with different attributed functions. In lung and pancreatic cancers, its expression correlates with poor prognosis. However, it has an anti-proliferative effect in colon and prostate malignancies. CEACAM1 expression in normal melanocytes is scant, but is increased with tumor progression [8], [9] and it is overexpressed in most cases of metastatic melanoma [10], [11]. CEACAM1 expression on melanoma cells protects them from an immune attack. As activated lymphocytes also express CEACAM1, its homophilic interactions between them and melanoma cells inhibit TIL mediated killing [12], [13]. We recently developed a novel approach for melanoma immunotherapy, based on a functional blocking of CEACAM1 with a specific mAb [10].

SOX9 (sex determining region Y [SRY]-related HMG-box 9) belongs to the SOX family, a conserved group of transcription factors sharing a high mobility group (HMG) domain for DNA binding [14]. SOX9 has a crucial role in the embryo, taking part in chondrogenesis and sex determination [15]. In normal melanocytes, SOX9 takes part in the signaling pathway following exposure to UVB, regulating microphthalmia-associated transcription factor (MITF) and tyrosinase expression [16]. Different functions have been attributed to SOX9 in several malignancies. It was shown to promote cell proliferation and tumorigenicity in lung adenocarcinoma, human glioma and colorectal cancer [17]-[19]. However, SOX9 inhibits tumor growth in endometrial and ovarian cancer cells [20], [21]. In melanoma, previous works demonstrated that overexpression of SOX9 inhibits growth of melanoma cells and causes cell cycle arrest [22], [23].

A connection between SOX9 and CEACAM1 was previously reported. In colon epithelial cells, SOX9 up-regulates CEACAM1 expression [24]. A different report shows an opposite correlation between SOX9 and CEACAM1 in Crohn's disease [25]. In this study we investigate the role of SOX9 in regulating CEACAM1 expression and thereby immune resistance in melanoma cells.

## RESULTS

### SOX9 affects CEACAM1 expression in melanoma cells

**Figure 1:**
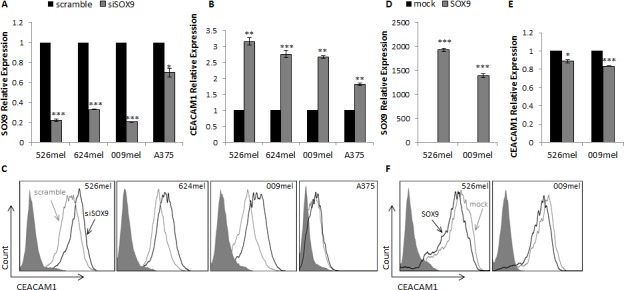
SOX9 influences CEACAM1 expression in melanoma cells **A.**-**C.** 526mel, 624mel, 009mel and A375 cells were transfected with an anti-SOX9 siRNA or a scrambled control. 72 hours post-transfection, SOX9 **A.** and CEACAM1 **B.** mRNA levels were assessed by qPCR and normalized to GAPDH. CEACAM1 protein levels were evaluated by flow Cytometry **C. D.**-**F.** 526mel and 009mel cells were transfected with a SOX9 construct or an empty vector (mock). 72 hours post-transfection, SOX9 **D.** and CEACAM1 **E.** mRNA levels were assessed by qPCR and normalized to GAPDH. CEACAM1 protein levels were evaluated by flow cytometry **F.**. Figures show a representative experiment out of several performed. Asterisks represent P values: *P < 0.05; **P < 0.01; ***P < 0.001 (2-tailed *t* test).

The effect of manipulations in SOX9 expression on CEACAM1 was tested in several melanoma cell cultures. SOX9 was efficiently silenced in all melanoma lines tested except for A375, using SOX9-specific siRNA as compared to control scrambled RNA sequence (Figure 1A). Accordingly, CEACAM1 was up-regulated in all melanoma lines both at the mRNA (Figure 1B) and protein (Figure 1C) levels, except for A375. It should be noted that the basal SOX9 expression level in A375 cells is about 200-300 times lower than in the 526mel, 624mel and 009mel cell lines (data not shown). This may account for inefficient silencing of SOX9 and hence no effect on CEACAM1 expression.

In a similar manner, overexpression of SOX9 caused down-regulation of CEACAM1 compared to mock-transfected cells (Figure 1D-1F). This effect was less prominent than in the knockdown of endogenous expression experiments.

These experiments suggest that SOX9 regulates the expression of CEACAM1.

To test for a possible correlation in SOX9 and CEACAM1 expression in melanoma cells, SOX9 and CEACAM1 mRNA levels were assessed by qPCR in 9 melanoma cell lines and 15 low passage metastatic melanoma cultures. No statistically significant correlation was observed between the mRNA expression levels (Supplementary Figure, 1), and the correlation coefficient was R = 0.40.

### SOX9 negatively regulates the CEACAM1 promoter in an indirect manner

**Figure 2:**
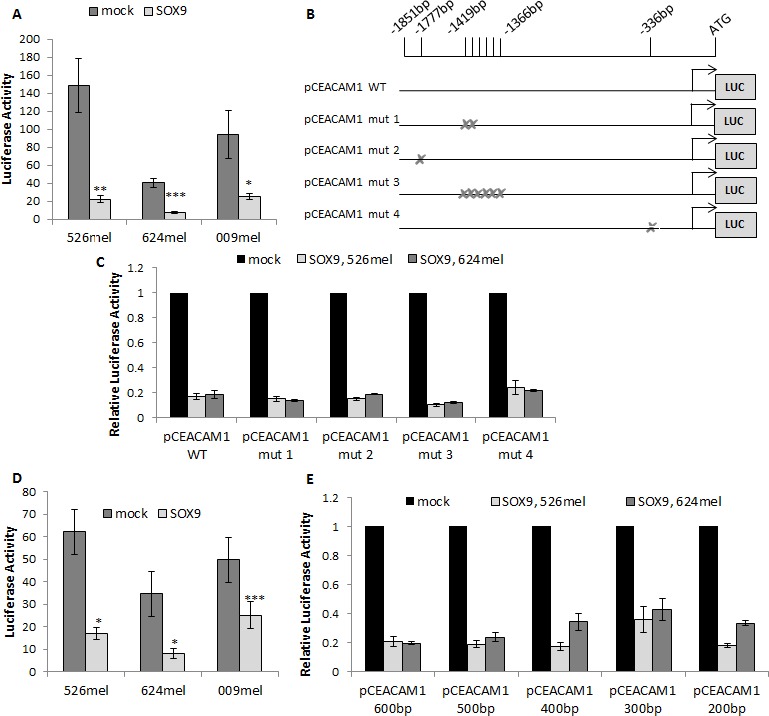
SOX9 negatively regulates CEACAM1 promoter in an indirect manner Dual luciferase reporter assays were performed with melanoma cells co-transfected with pCEACAM1 and with SOX9 overexpression or an empty vector (mock). Luciferase activity was measured and normalized to Renilla activity. Experiments were performed in sixplicates. **A.** Dual luciferase reporter assay performed with CEACAM1 long promoter segment (~1900bp). Data represent the mean ± SEM of 6 independent experiments. **B.**-**C.** Dual luciferase reporter assays were performed with melanoma cells co-transfected with pCEACAM1 wild-type (WT) or mutated pCEACAM1 and with SOX9 overexpression or an empty vector. Relative luciferase activity was normalized to the luciferase activity of control vector. **D.** Dual luciferase reporter performed with CEACAM1 short promoter segment (~600bp). Data represent the mean ± SEM of 6 independent experiments. **E.** Dual luciferase reporter assays performed with truncated segments of pCEACAM1. Results shown are of a representative experiment out of 3 performed. Asterisks represent P values: *P < 0.05; **P < 0.01; ***P < 0.001 (2-tailed *t* test).

Manipulations in SOX9 expression alter CEACAM1 at the mRNA and protein levels, implying on transcriptional regulation. The effect of SOX9 on the activity of the full CEACAM1 putative promoter (~1900bp upstream to ATG start codon) was tested in luciferase reporter assays. The luciferase reporter construct was co-transfected with SOX9 or with an empty vector as a control into different melanoma lines. SOX9 induced a remarkable inhibition in luciferase activity, as compared to control, in all melanoma cell lines tested (Figure 2A). In order to identify the binding site(s) for SOX9 within the CEACAM1 promoter, we employed three sources: a) two binding sites for SOX9 within the CEACAM1 promoter were depicted in a previous report focusing on colon epithelium [24]. Notably, this paper showed an opposite effect, as overexpression of SOX9 caused up-regulation of CEACAM1 expression; b) locating the SOX core-binding element (SCBE) - AACAAT [26] within the CEACAM1 promoter; c) use of the MAPPER_2_ database [27], an analysis tool for finding patterns and regulation elements in the DNA. In total, eight possible binding sites were identified. Four pCEACAM1 constructs carrying deletions in different combinations of putative binding sites were established (Figure 2B). Surprisingly, none of the deletions had any impact on the suppressive effect of SOX9 on the CEACAM1 promoter in two different melanoma cell lines (Figure 2C). This finding contradicts the previous report in colon epithelial cells [24]. We therefore hypothesized that SOX9 regulates the activity of the CEACAM1 promoter indirectly.

### SOX9 regulates CEACAM1 primarily *via* Sp1 and ETS1

In order to narrow down the area on which SOX9 exerts its effect within the CEACAM1 promoter, a shorter fragment of the promoter was cloned, 600bp upstream to ATG start codon. The shorter construct was still similarly inhibited by SOX9, as tested in luciferase reporter assays in three melanoma cell lines (Figure 2D). Additional promoter constructs were cloned, each shorter by 100bp, down to a minimum of 200bp upstream to the ATG start codon. Importantly, the inhibitory effect of SOX9 was unaffected and still strongly evident even in the shortest segment (Figure 2E). These results imply that SOX9 affects mainly the proximal 200bp of the promoter. MAPPER_2_ database search for transcription factors that bind to the proximal 200bp segment of the CEACAM1 promoter highlighted putative binding sites for three major transcription factors that could act as mediators: Sp1 (one site), ETS1 (four sites) and AP-2 (one site).

**Figure 3:**
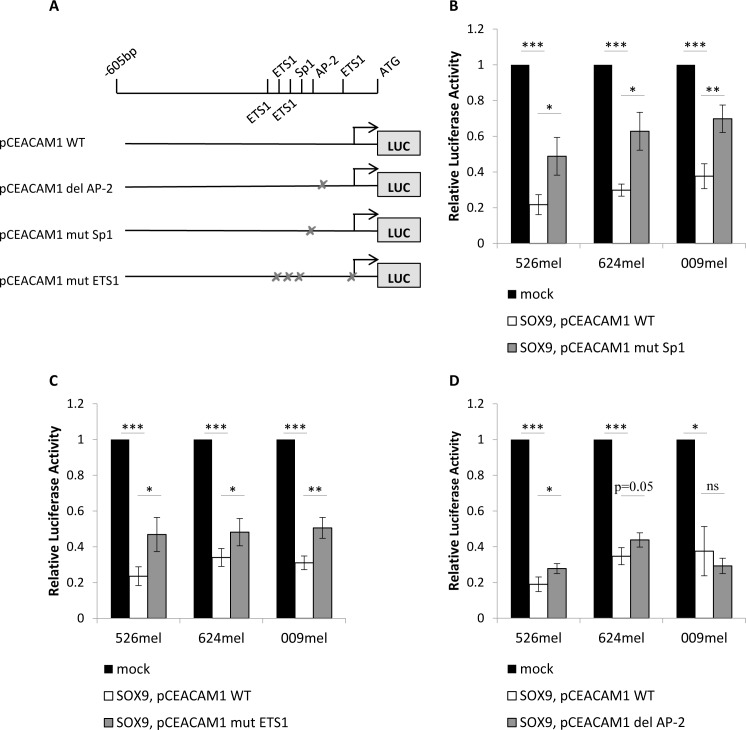
Transcription factors Sp1, ETS1 and AP-2 mediate the SOX9 down-regulation of the CEACAM1 promoter Dual luciferase reporter assays were performed with melanoma cells co-transfected with pCEACAM1 wild-type (WT) or mutated pCEACAM1, and with SOX9 overexpression or an empty vector (mock). Luciferase activity was measured and normalized to Renilla activity. Relative luciferase activity was normalized to the luciferase activity of control vector. Experiments were performed in sixplicates. **A.** Scheme of mutated constructs of the transcription factors' putative binding sites. **B.** Experiments with mutation of the Sp1 putative binding site **C.** Experiments with mutations of four ETS1 putative binding sites **D.** Experiments with deletion of the AP-2 putative binding site. Data in all figures represent the mean ± SEM of at least 3 independent experiments. Asterisks represent P values: *P < 0.05; **P < 0.01; ***P < 0.001 (2-tailed *t* test).

A series of point mutations or deletions of the putative binding sites for each of these transcription factors was generated based on the 600bp promoter, as described in Figure 3A. Luciferase reporter assays were repeated with the mutated or wild-type (WT) pCEACAM1 constructs, which were co-transfected with SOX9 or an empty vector, in three melanoma cell lines. The suppressive effect of SOX9 on the promoter was significantly hindered in the construct bearing the mutated Sp1 binding site, in all three melanoma lines (Figure 3B). A similar, yet milder abrogative effect was observed with the construct bearing the mutated ETS1 binding sites (Figure 3C). Deletion of the AP-2 binding site had a marginal effect in two of the three melanoma lines examined (Figure 3D). These combined results suggest that SOX9 mediates its suppressive effect on the CEACAM1 promoter primarily *via* Sp1 and partly *via* ETS1.

### SOX9 creates a complex with Sp1

**Figure 4:**
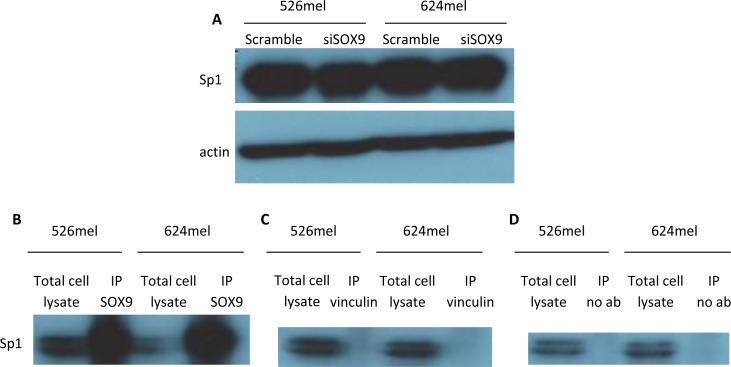
SOX9 does not alter Sp1 expression, but physically interacts with Sp1 in melanoma cells **A.** 526mel and 624mel cells were transfected with an anti-SOX9 siRNA or a scrambled control. 72 hours post-transfection, proteins were extracted from cells and Sp1 expression was evaluated *via* Western blot. **B.**-**D.** 526mel and 624mel cell lysates were immunoprecipitated with an anti-SOX9 antibody **B.** Anti-vinculin antibody **C.** or no antibody **D.** served as negative controls. Cell lysates and immunoprecipitates were analyzed by immunoblotting for Sp1. All blots show one representative experiment out of 3 performed.

The putative Sp1 binding site in the CEACAM1 promoter is chiefly involved in mediating CEACAM1 down-regulation by SOX9 (Figure 3B). Knockdown of SOX9 had no significant effect on the expression level of Sp1 (Figure 4A), implying on other mechanisms such as physical protein-protein interactions. It is established that Sp1 forms complexes with other proteins to mediate its transcriptional activity [28]. It was previously reported that SOX9 and Sp1 may form functional complexes that up-regulate type II collagen expression [29], [30]. In line with this data, co-immunoprecipitation of SOX9 with Sp1 in two melanoma cell lines confirms that Sp1 physically binds to SOX9 in melanoma cells (Figure 4B). Western blotting for Sp1 was negative following immunoprecipitation of the negative controls vinculin (Figure 4C) or without any antibodies (Figure 4D). The collective evidence supports a possible mechanism by which SOX9 and Sp1 regulate the CEACAM1 promoter as a complex.

### SOX9 alters the expression of ETS1

**Figure 5:**
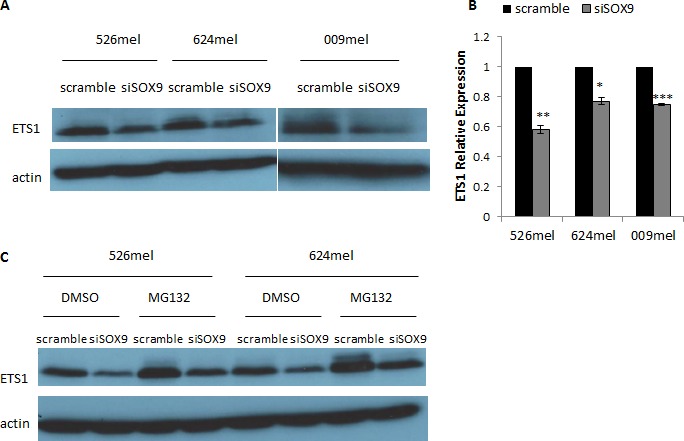
SOX9 alters ETS1 expression in melanoma cells **A.**-**B.** 526mel, 624mel and 009mel cells were transfected with an anti-SOX9 siRNA or a scrambled control. 72 hours post-transfection, protein and total RNA were extracted from cells. ETS1 protein levels were evaluated by Western Blot **A.**, and mRNA levels were assessed by qPCR and normalized to GAPDH **B.**. **C.** 526mel and 624mel cells transfected with an anti-SOX9 siRNA or a scrambled control were treated with proteasome inhibitor MG132 or DMSO for 6 hours. 72 hours post-transfection, proteins were extracted from cells and ETS1 expression was evaluated by Western Blot. Figures show a representative experiment. Asterisks represent P values: *P < 0.05; **P < 0.01; ***P < 0.001 (2-tailed *t* test).

Luciferase reporter assay experiments pointed on the involvement of ETS1 in the regulation of CEACAM1 by SOX9, though to a lesser extent than Sp1 (Figure 3). Knockdown of SOX9 had no effect on the expression of Sp1 (Figure 4A), but significantly down-regulated ETS1 expression (Figure 5A). Notably, this effect was very moderate at the mRNA level (Figure 5B), suggesting regulation at the translational or post-translational levels. Proteasome inhibition with MG132 resulted in an increased amount of ETS1 protein in the MG132-treated cells in comparison to DMSO-treated cells (Figure 5C). However, the ETS1 expression ratio between cells treated with siRNA for SOX9 or a scrambled sequence was not affected by MG132 (Figure 5C). This means that SOX9-mediated down-regulation of ETS1 at the protein level is unlikely to be facilitated by proteasome-mediated degradation. Similar results were observed in two melanoma cell lines (Figure 5C).

### SOX9 silencing increases melanoma cells immune resistance

**Figure 6:**
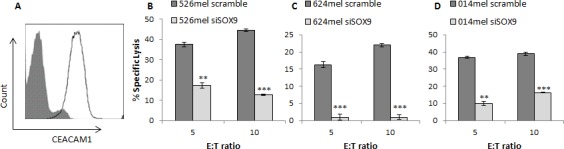
SOX9 regulates immune resistance in melanoma cells **A.** Expression of CEACAM1 in TIL14, as assessed by flow cytometry. **B.**-**D.** 526mel **B.**, 624mel **C.** and 009mel **D.** melanoma cells transfected with an anti-SOX9 siRNA or a scrambled control were incubated with effector cells TIL14. Following incubation, cells were stained with PI and specific lysis was assessed using flow cytometry. Experiments were performed in triplicates. Figures show one representative experiment for each cell type out of three performed. Asterisks represent *P* values: *P < 0.05; **P < 0.01; ***P < 0.001 (2-tailed *t* test).

SOX9 expression was selectively silenced in several melanoma cells with an anti-SOX9 siRNA or a scrambled sequence as a control. SOX9-manipulated melanoma cell lines were used as target cells in cytotoxicity assays. The bulk primary TIL cells (TIL14) were used as effectors in different effector to target (E:T) ratios. TIL14 cells express CEACAM1 at high level (Figure 6A), allowing for CEACAM1 homophilic interactions with melanoma cells. Remarkably, silencing of SOX9 rendered all tested melanoma lines significantly more resistant to killing by TIL14 cells, as compared to the control (Figure 6B-6C). Similar results were observed also when the autologous 014mel cells, which were derived from the same patient as TIL14, were tested (Figure 6D). CEACAM1 expression was up-regulated in the SOX9-silenced cells to a similar extent as depicted in Figure 1 (data not shown). These results, combined with the known role of CEACAM1 in protecting melanoma cells from an immune attack [10], [12], confirm the role of SOX9 in immune evasion, *via* CEACAM1 expression.

## DISCUSSION

It is well known that melanoma is an immunogenic tumor. Melanoma cells express a variety of tumor associated antigens and contain the highest DNA mutation load [2], [3], instigating an adaptive immune response and production of specific anti-tumor T cells [4].

Our lab has previously shown that CEACAM1, a transmembrane glycoprotein abundantly expressed in most metastatic melanomas but not in normal melanocytes, is a key factor in the immune interaction between melanoma cells and activated lymphocytes. Furthermore, it is expressed on activated T-lymphocytes, enabling a homophilic interaction that inhibits T-cell mediated killing. Thus, CEACAM1 expressed on metastatic melanoma cells protects them from an immune attack, and CEACAM1 blockade renders the cells more susceptible to T cells [10], [12], [13]. Delineation of CEACAM1 regulation mechanisms in melanoma cells is therefore of clear importance. Here we focus on the transcription factor SOX9, and show that it influences CEACAM1 expression and immune resistance in melanoma cells.

A connection between SOX9 and CEACAM1 was previously reported but results were contradictory. Zalzali et al reported in colon epithelial cells that SOX9 up-regulates CEACAM1 expression [24]. Roda et al showed a correlation between SOX9 and CEACAM1 in Crohn's disease, but in the opposite direction [25]. Here we show in several melanoma cell lines that overexpression or knockdown of SOX9 causes down-regulation or up-regulation of CEACAM1, respectively, in mRNA and protein levels (Figure 1). The effect of SOX9 overexpression was less prominent than the one noted in knockdown of endogenous SOX9 expression, suggesting that the role of SOX9 is within its physiological expression range. SOX9 and CEACAM1 mRNA levels were evaluated in 24 melanoma cell cultures to test for a possible correlation (Supplementary Figure 1). No correlation was detected. This is not unexpected, as we show the regulation of CEACAM1 by SOX9 to be indirect.

Since SOX9 has a known role as a transcription factor, we set out to examine whether it affects CEACAM1 promoter activity. Indeed, luciferase reporter assays show that SOX9 down-regulates CEACAM1 at a transcriptional level (Figure 2A). Zalzali et al showed that SOX9 increases CEACAM1 transcription in a direct manner, *via* two specific binding sites [24]. However, our results not only show an opposite regulation, but also that SOX9 does not down-regulate CEACAM1 promoter directly. Deletion of all eight possible binding sites, among them the two sites depicted in the aforementioned report, did not diminish the effect of SOX9 on the CEACAM1 promoter (Figure 2B-2C). Therefore, we infer that SOX9 regulates CEACAM1 in an indirect manner. This double discrepancy between our results and the previous report [24], where it was demonstrated that SOX9 directly binds to the CEACAM1 promoter to activate it, may stem from the different cells used in the experiments, and the different nuclear milieu in those cells. Other factors in the nuclei of melanoma cells could compete with SOX9 for the same binding sites, or bind to close areas in the DNA and create a steric interference. It is also worth noting that the role of CEACAM1 in colon epithelium is distinctly different than in melanoma. In melanoma, CEACAM1 promotes tumor aggressiveness, whereas in colon carcinoma it acts as a tumor growth suppressor and is down-regulated in the early phases of tumor development [31], [32].

Luciferase assays with truncated segments of the CEACAM1 promoter showed that SOX9 exerts its effect in the proximal 200bp portion (Figure 2E). Our hypothesis was that a second transcription factor mediates the effect of SOX9 over CEACAM1. We searched for major transcription factors that have single or multiple binding sites in the 200bp proximal segment of the CEACAM1 promoter. Bioinformatics analysis with the MAPPER_2_ database, crossing with PubMed database, concluded in three possible transcription factors - AP-2 [33], Sp1 [29], [30], [34] and ETS1 [35]-[37]. Indeed, luciferase reporter assay experiments with mutated pCEACAM1 constructs, containing deletions or mutations of the transcription factors' putative binding sites, suggest that all three transcriptions factors are involved in the regulation, though to different extents (Figure 3). Each of the mutated constructs partially negated the down-regulation of the CEACAM1 promoter by SOX9. This partial abrogation was most substantial by Sp1, intermediate by ETS1 and negligible by AP-2. As the effect of AP-2 putative binding site deletion was marginal, we did not continue to pursue this angle in our research.

Previous studies have exhibited a functional role for Sp1 in melanoma cells. Sp1 influences invasiveness of melanoma cells by regulating cathepsin B [38] and metalloproteinase MT1-MMP expression [39]. It also takes part in regulation of angiogenesis by affecting key factors, such as VEGF and TNF-alpha [40]. Sp1 has been shown to bind to the CEACAM1 promoter and activate it in human breast and colon epithelial cells [41], [42]. Notably, our data indicates that in melanoma cells Sp1 is involved in deactivation of the CEACAM1 promoter (Figure 3B). Controlled manipulation of SOX9 failed to alter Sp1 expression level (Figure 4A). Sp1 operates in many systems as a complex with other proteins [28]. Two previous papers revealed that SOX9 creates a complex with Sp1 to activate the type II collagen promoter in articular chondrocytes [29], [30]. Indeed, co-immunoprecipitation experiments in several melanoma lines showed that Sp1 physically binds to SOX9 (Figure 4B-4D). Together with the luciferase reporter assay experiments, these results could indicate that SOX9 and Sp1 create a complex that regulates CEACAM1 promoter activity in a joint mechanism. However, while in chondrocytes the SOX9-Sp1 complex activates type II collagen promoter, here we show this complex inhibits the CEACAM1 promoter. This could be explained by the binding of additional factors to this complex, such as transcription regulators and chromatic remodeling factors. These factors may differ between melanoma cells and chondrocytes, as their identity is dependent on the cell-specific nuclear milieu.

The role of ETS1 has also been explored in melanoma. Its expression increases as the disease progresses [43]. ETS1 regulates key factors that promote melanoma development and survival. These factors include MET, a proto-oncogene often overexpressed in melanoma cells, which promotes cell growth [44]. Another target gene is c-Jun, a key player in tumor development [45]. ETS1 also regulates genes involved in melanoma cell invasiveness: MMP1, MMP3 and integrin-β3 [46]. To our knowledge, there is no current data regarding possible regulation of CEACAM1 by ETS1.

Mutations in the putative binding sites of ETS1 in the CEACAM1 promoter partially negated the SOX9 down-regulation of its activity, suggesting that ETS1 also has an inhibiting effect on CEACAM1 promoter. Next, we aimed to investigate a potential connection between SOX9 and ETS1. We assessed ETS1 protein and mRNA expression in melanoma cells after SOX9 silencing. SOX9 knockdown cells exhibited lower levels of ETS1 protein and mRNA in comparison with control cells (Figure 5A-5B). This down-regulation of ETS1 expression by SOX9 fits our hypothesis: SOX9 overexpression causes ETS1 up-regulation, which in turn down-regulates CEACAM1 promoter activity. In addition, our results also showed that this down-regulation of ETS1 expression is distinct in the protein level, though changes in the mRNA levels were moderate. This discrepancy suggests a regulation at translational or post-translational levels. Treatment with MG132, a proteasome inhibitor, did not change the down-regulation of ETS1 caused by SOX9 silencing (Figure 5C), suggesting this is not the mechanism by which SOX9 influences ETS1 protein expression. Another possible mechanism may be related to regulation of miRNAs by SOX9, as miRNAs can bind the 3′UTR segment of ETS1 mRNA and inhibit its translation. Previous papers describing a relation between SOX9 and ETS1 are scarce. Betancur et al showed that SOX9 and ETS1 regulate SOX10 expression together in a synergistic manner in the neural crest of chicken embryo [35], [36]. Gao et al demonstrated that SOX9 is up-regulated in the neural crest of ETS1-deficient mice [37]. To our knowledge, our results are the first description of a possible regulation of ETS1 by SOX9.

Finally, functional studies show that knockdown of SOX9 renders melanoma cells more resistant to TIL-mediated killing (Figure 6). This correlates with the subsequent up-regulation of CEACAM1 and its known role in protecting melanoma cells from an immune attack. These results were observed in several melanoma cell cultures, suggesting this is not a cell line limited phenomenon. To our knowledge, this is the first time that SOX9 is investigated in an immunological context. In future studies, it would be interesting to test the relevance of SOX9 expression in predicting response to treatment with immunotherapeutic agents.

## MATERIALS AND METHODS

### Cells

Human metastatic melanoma cell lines 526mel and 624mel were obtained from Dr. Steve Rosenberg (National Cancer Institute, Bethesda, MD, USA). 009mel and 014mel are primary cultures derived from surgically removed metastatic melanoma specimens of patients 009 and 14, respectively. TIL14 bulk culture was established from a specimen of metastatic melanoma of patient 14 [47], obtained according to Israel Ministry of Health approval no. 3518/2004. Melanoma cultures were grown in RPMI-1640 medium (Biological Industries, Beit Ha-Emek, Israel) supplemented with 10% FBS, 100 μg/ml Pen/Strep, 2mM L-glutamine, 25mM Hepes and 1mM sodium-pyruvate (Biological Industries, Beit Ha-Emek, Israel). The A375 human metastatic melanoma cell line (ATCC, Manassas, VA, USA) was cultured in DMEM medium (Biological Industries, Beit Ha-Emek, Israel) supplemented with 10% FBS, 100 μg/ml Pen/Strep, 2mM L-glutamine, 1mM sodium-pyruvate and non-essential amino-acids (Biological Industries, Beit Ha-Emek, Israel). TIL14 bulk cultures were grown as previously described [12].

### Knockdown of SOX9

siRNA-mediated gene knockdown Trilencer-27siRNA kit (OriGene Technologies Inc, Rockville, MD, USA) was used. 1.0-1.5×10^5^ cells were seeded per well in 6-well plates, and transfected with either SOX9-specific siRNA (15nM) or a scrambled control (15nM), using JetPRIME transfection reagent (Polyplus-transfection SA, Illkirch, France), according to manufacturer's instructions.

### Cloning, point mutations and deletions

SOX9 expression vector was purchased from the plasmID Repository at Harvard Medical School (clone HsCD00004049). SOX9 cDNA was amplified from this vector and inserted into a pcDNA3 expression vector (Invitrogen, Carlsbad, CA, USA) using HindIII and XhoI restriction enzymes (New England Biolabs, Ipswich, MA, USA). DNA from melanoma cells for cloning of the CEACAM1 promoter was purified using GenElute Mammalian Genomic DNA Miniprep Kit (Sigma-Aldrich, St. Louis, MO, USA). Promoter fragments containing the full or partial putative promoter of CEACAM1 were amplified and cloned into pGL4.14 reporter vector (Promega, Madison, WI, USA) using XhoI and HindIII sites. Point mutations and deletions were introduced into the various constructs using specific primers, DNA synthesis with KOD Hot Start Polymerase (Merck Millipore, Darmstadt, Germany) and ultimately DpnI (New England Biolabs, Ipswich, MA, USA) digestion at 37°C for 1 hour. The full sequences of all primers used are detailed in Supplementary Table 1.

### Overexpression of SOX9

1.5×10^5^ melanoma cells were seeded per well in 6-well plates. Transient transfections of pcDNA3/SOX9 or an empty vector were performed using TurboFect Transfection Reagent (Thermo Scientific, Waltham, MA, USA) according to manufacturer's instructions.

### RNA isolation and reverse transcription

Total RNA was isolated using TRI reagent (Sigma-Aldrich, St. Louis, MO, USA) according to manufacturer's instructions. cDNA was generated by Transcriptor Universal cDNA Master (Roche, Penzberg, Germany).

### Quantitative real-time PCR

Primers for different genes were designed using the Primer-Express software (Applied Biosystems, Foster City, CA). The full sequences of all primers used are depicted in Supplementary Table 1. qPCR reactions were performed in triplicates on LightCycler480 system (Roche, Penzberg, Germany). Gene transcripts were detected using LightCycler480 SYBR Green I Master (Roche, Penzberg, Germany) and gene-specific primers, according to manufacturer's instructions. Reactions were normalized to GAPDH endogenous control.

### Luciferase reporter assay

1×10^4^ melanoma cells were seeded per well in 96-well plates, and co-transfected using TurboFect Transfection Reagent (Thermo Scientific, Waltham, MA, USA) with pCEACAM1 constructs or an empty pGL4.14 vector, and pcDNA3/SOX9 or an empty pcDNA3 vector and pRL Renilla luciferase reporter vector (Promega, Madison, WI, USA). After 48 hours, cells were lysed and firefly luciferase activity was measured with Dual Luciferase Reporter Assay System (Promega, Madison, WI, USA) and normalized to Renilla.

### Flow cytometry

MRG1, a homemade specific to CEACAM1 monoclonal mouse antibody [10], was used to determine surface CEACAM1 expression. 1×10^5^ cells were incubated with 0.1μg of antibody diluted in PBS/ 1% EDTA/ 0.5% bovine serum albumin (BSA)/0.05% sodium-azide [fluorescence-activated cell-sorting (FACS) medium] for 30 minutes on ice. Cells were centrifuged at 500 x g for 5 minutes and supernatant was removed. Cells were then incubated for 30 minutes on ice with a secondary Alexa Fluor 488 goat anti-mouse IgG antibody (Life Technologies, Carlsbad, CA, USA), washed with FACS medium, and analyzed with FACSCalibur instrument (BD Biosciences, San Jose, CA, USA) and FlowJo software (Tree Star Inc., Ashland, OR, USA).

### Co-immunoprecipitation and western blotting

5×10^6^ cells were cross-linked with DSP (Thermo Scientific, Waltham, MA, USA) and lysed in radio immunoprecipitation assay buffer (RIPA) lysis buffer (Sigma-Aldrich, St. Louis, MO, USA) supplemented with protease inhibitor cocktail (Roche, Penzberg, Germany) on ice for 20 minutes. Insoluble material was removed by centrifugation at 14,000 rpm for 15 minutes at 4°C. Surebeads magnetic beads (Bio-rad, Hercules, CA, USA) were incubated for 10 minutes in rotation with either anti-SOX9 (#ab3697, Abcam, Cambridge, UK) or anti-vinculin (#ab129002, Abcam, Cambridge, UK) antibodies or no antibody, and then washed three times with PBS-T (PBS + 0.1% tween 20). Then, cell lysates were added to the beads for rotation overnight at 4°C. Following incubation, the beads-antibody-target protein complex was washed with PBS-T three times, and eluted with 1xLaemmli buffer (Bio-rad, Hercules, CA, USA) at 70°C for 10 minutes. Both total cell lysates and immunoprecipitates were analyzed by SDS-PAGE. Western blot using anti-SP1 (#07-645, Merck Millipore, Darmstadt, Germany), anti-ETS1 (#ab10936, Abcam, Cambridge, UK) or anti- β-actin (#MAB1501, Merck Millipore, Darmstadt, Germany) specific antibodies was performed according to standard protocol, and was developed with ECL reaction, as previously described [48].

### Cytotoxicity assay

Cytotoxicity measurements based on carboxy-fluorescein succinimidyl ester (CFSE)-labeling of target cells and co-staining with propidium iodide (PI) after incubation with effector cells, were performed using flow cytometry, as previously described [13].

### Statistics

Data were analyzed using the parametric unpaired two-tailed Student's *t* test. In all graphs, error bars represent Standard Error. Asterisks indicate P values: * *P* < 0.05, ** *P* < 0.01, *** *P* < 0.001.

## SUPPLEMENTARY MATERIAL TABLE AND FIGURE


