# Supplementary material for: SOX9 indirectly regulates CEACAM1 expression and immune resistance in melanoma cells
Source: Oncotarget. 2016 Feb 14;7(21):30166–77. doi: 10.18632/oncotarget.7379 (PMC5058672; doi:10.18632/oncotarget.7379)
Supplement: Supplementary file 1 [file oncotarget-07-30166-s001.pdf]

SOX9 indirectly regulates CEACAM1 expression and immune resistance in melanoma cells

Supplementary Material

Supplementary Table 1: Primers

Real-time primers

Gene	Forward primer	Reverse Primer
SOX9	5'- CCCTATCGACTTCCGCGAC	5'-TCGTTGACATCGAAGGTCTCG
CEACAM1	5'- GAGTAGTGGCCCTGGTTGCTC	5'- CGCTGGTCGCTTGCCCT
ETS1	5'- CTGACCCAGATGAGGTG	5'- CGCTGTCTTGTGGATGAT
GAPDH	5'- TGCACCACCAACTGCTTAGC	5'- CGCATGGACTGTGGTCATGAG

Cloning primers

Insert	Forward primer	Reverse Primer
SOX9 cDNA	5'- CATGAATCTCCTGGACCCC	5'- TCAAGGTCGAGTGAGCTGT
pCEACAM1 full	5'- GTTCCAGTGATTCTCCTGC	5'- GGTGTCTCCTGCTGGC
pCEACAM1 600bp	5'- CTGAGACCCCTGGACTTGGGTCT	5'- GGTGTCTCCTGCTGGCCCTGT
pCEACAM1 500bp	5'- CCACCTCTGTCACCTTCC	5'- GGTGTCTCCTGCTGGC
pCEACAM1 400bp	5'- GGATATGCCAGGGTTCTCT	5'- GGTGTCTCCTGCTGGC
pCEACAM1 300bp	5'- CTGATGGGGACAGAGGTC	5'- GGTGTCTCCTGCTGGC
pCEACAM1 200bp	5'- AGGACAAAGGTAGGAATGAGAG	5'- GGTGTCTCCTGCTGGC

Mutations / deletions primers

	Position	Location upstream to start codon	Primers
Deletion of SOX9 site 1 in pCEACAM1	Chr19, 42530146 - 42530151	(-1777) - (-1772) bp	Fw: 5'- GACCACACCTGGCTAATTTTTTT TAGTAGAGACGGGG
			Rev: 5'- CCCCGTCTCTACTAAAAAAATT AGCCAGGTGTGGTC
Deletion of SOX9 sites 2 & 3 in pCEACAM1	Chr19, 42529777 - 42529793	(-1419) - (-1403) bp	Fw: 5'- ATTCTCAAGCCCCCAAAGGCACT TTTTGTTTTGTTTTGTTTTTGC
			Rev: 5'- GCAAAAACAAAACAAAACAAAA AGTGCCTTTGGGGGCTTGAGAAT
Deletion of SOX9 sites 4-7 in pCEACAM1	Chr19, 42529740 - 42529776	(-1402) - (-1366) bp	Fw: 5'- GCCCCCAAAGGCACCCTCTGGG ACAGAT
			Rev: 5'- ATCTGTCCCAGAGGGTGCCTTTG GGGGC
Deletion of SOX9 site 8 in pCEACAM1	Chr19, 42528704 - 42528710	(-336) - (-330) bp	Fw: 5'- CCCATAGCCAGGACAGGTCAGT GCTGGA
			Rev: 5'- TCCAGCACTGACCTGTCCTGGCT ATGGG
Mutation of Sp1 site in	Chr19,	(-165) - (-152) bp	Fw: 5'- AATGAGAGGAGAGGAAACAGA GCTTCCTGGACAGTAACCGAACCAGCACACA

pCEACAM1	42528526 - 42528539		TGATCAG
			Rev: 5’- CTGATCATGTGTGCTGGTTCGGTTAC TGTCCAGGAAGCTCTGTTTCCTCTCCTCTCATT
Mutation of ETS1 site 1 in pCEACAM1	Chr19, 42528569 - 42528565	(-199) - (-195) bp	Fw: 5’- AGAGAAAGTAAGGACAAAGGT AAAAATGAGAGGAGAGGAAACAGAG
			Rev: 5’- CTCTGTTTCCTCTCCTCTCATTTT TACCTTTGTCCTTACTTTCTCT
Mutation of ETS1 site 2 in pCEACAM1	Chr19, 42528555 - 42528559	(-185) - (-181) bp	Fw: 5’- CAAAGGTAAAAATGAGAGGA GAAAAAACAGAGCTTCCTGGACAAAC
			Rev: 5’- GTTTGTCCAGGAAGCTCTGTTTT TTCTCCTCTCATTTTTACCTTTG
Mutation of ETS1 site 3 in pCEACAM1	Chr19, 42528543 - 42528547	(-173) - (-169) bp	Fw: 5’- AAAATGAGAGGAGAAAAAAC AGAGCTTTTTGGACAAACCCCGC
			Rev: 5’- GCGGGGTTTGTCCAAAAAGCTC TGTTTTTTCTCCTCTCATTTT
Mutation of ETS1 site 4 in pCEACAM1	Chr19, 42528425 - 42528429	(-55) - (-51) bp	Fw: 5’- CAGCCGTGCTCGAAGCGTTTTTG GAGCCCAAG
			Rev: 5’- CTTGGGCTCCAAAAACGCTTCGA GCACGGCTG
Deletion of AP-2 binding site in pCEACAM1	Chr19, 42528485 - 42528500	(-126) - (-111) bp	Fw: 5’- GATCAGACAAAGCTCTGCTCAG CACAGAGAGTGG
			Rev: 5’- CCACTCTCTGTGCTGAGCAGAGC TTTGTCTGATC

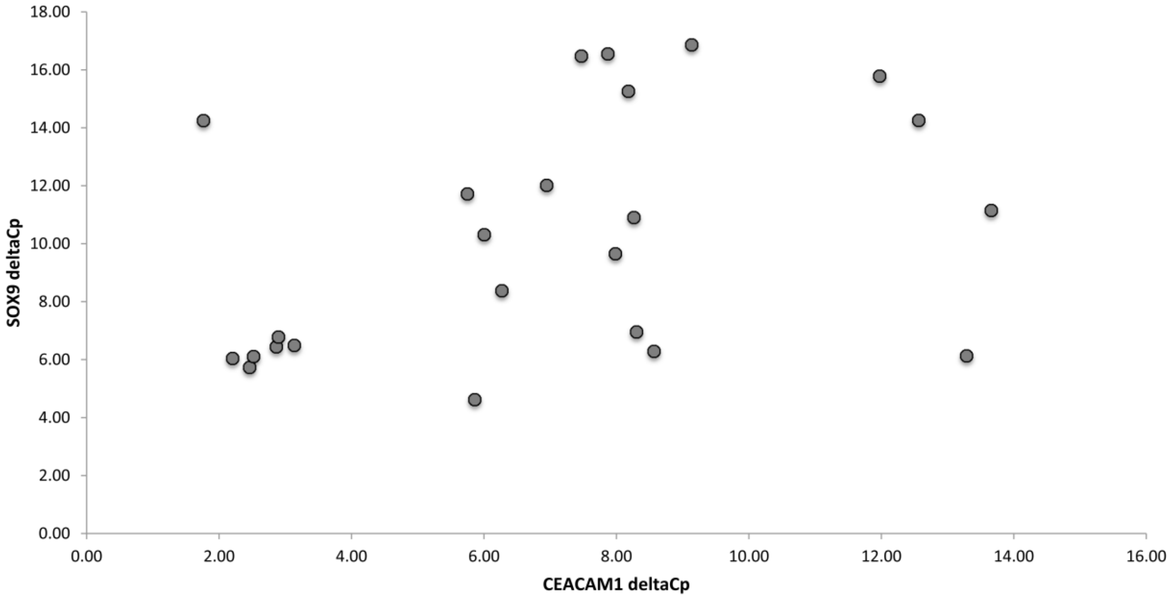

Figure 1: SOX9 and CEACAM1 mRNA expression in melanoma cell cultures. SOX9 and CEACAM1 mRNA expression in melanoma cell lines and low passage metastatic melanoma cultures, as determined by qPCR. Data represent one experiment performed in triplicates.
